# Recommendations for headache service organisation and delivery in Europe

**DOI:** 10.1007/s10194-011-0320-x

**Published:** 2011-03-05

**Authors:** T. J. Steiner, F. Antonaci, R. Jensen, M. J. A. Lainez, M. Lanteri-Minet, D. Valade

**Affiliations:** 1 Department of Neuroscience, Norwegian University of Science and Technology, Trondheim, Norway; 2 Department of Clinical Neuroscience, Imperial College London, St Dunstan’s Road, London, W6 8RP UK; 3 University Consortium for Adaptive Disorders and Headache (UCADH), Pavia, Italy; 4 Headache Medicine Centre, Department of Neurology, Policlinic of Monza, Monza, Italy; 5 Danish Headache Centre, Department of Neurology, University of Copenhagen, Glostrup, Denmark; 6 Department of Neurology, University Clinic Hospital, Valencia University, Valencia, Spain; 7 Département d’évaluation et traitement de la douleur, Pôle des neurosciences cliniques, CHU de Nice, Hôpital Pasteur, Nice, France; 8 Centre d’Urgence des Céphalées, Hôpital Lariboisière, Paris, France

**Keywords:** European Headache Federation, Global Campaign against Headache, Guidelines, Headache disorders, Service delivery and organisation

## Abstract

Headache disorders are a major public-health priority, and there is pressing need for effective solutions to them. Better health care for headache—and ready access to it—are central to these solutions; therefore, the organisation of headache-related services within the health systems of Europe becomes an important focus. These recommendations are the result of collaboration between the European Headache Federation and *Lifting The Burden*: the Global Campaign against Headache. The process of development included wide consultation. To meet the very high level of need for headache care both effectively and efficiently, the recommendations formulate a basic three-level model of health-care organisation rationally spread across primary and secondary health-care sectors, taking account of the different skills and expertise in these sectors. They recognise that health services are differently structured in countries throughout Europe, and not always adequately resourced. Therefore, they aim to be adaptable to suit these differences. They are set out in five sections: needs assessment, description of the model, adaptation, standards and educational implications.

## Introduction

The mission statement of the European Headache Federation (EHF) sets out its primary purpose: to improve life for those affected by headache disorders in Europe [1]. EHF undertakes a range of activities in pursuit of this aim. “Educating Europe” about headache—its nature, prevalence, causes, consequences and management—is of highest importance. With knowledge of headache, and especially these aspects of it, comes recognition of headache disorders as a major public-health priority, and awareness of the need for effective solutions to them.

European Headache Federation is also much concerned with what these solutions should be, and how they might be implemented. Since better health care for headache and ready access to it are their essence, the organisation of headache-related services within the health systems of Europe becomes an important priority also to maximise both effectiveness and cost-effectiveness. These recommendations are the result of collaboration between EHF and *Lifting The Burden* (LTB), the Global Campaign against Headache [2, 3].

Headache disorders are amongst the top ten causes of disability in Europe [4]. Three of these (migraine, tension-type headache and medication-overuse headache) have major significance for public health and health-service policy because they are common and responsible for almost all headache-related burden. The principal objective of headache services within a health-care system must be to mitigate this burden; their focus must be these three disorders.

Other headaches, although generally much less common, are nonetheless important as they may be symptoms of underlying disorders that threaten health and well being. These secondary headaches call for correct diagnosis and effective treatment, which sometimes are required urgently to prevent serious consequences. Management of these is, essentially, treatment of the causative disorder, and therefore arguably belongs outside headache services. On the other hand, their recognition must be the responsibility of the services to which affected patients present; where headache is the symptom, this is likely to be headache services, which must make adequate provision for them also.

## Purpose

Our aim was to formulate a basic model of health-care organisation rationally spread across primary and secondary health-care sectors and taking due account of the different skills and levels of expertise in these sectors.

We recognised, and endeavoured also to take into account, that health services are differently structured in countries throughout Europe, and not always adequately resourced.

The purpose of these recommendations is therefore to describe, and explain, a model for headache service organisation that

- meets the very high level of need for headache-related health care both effectively and efficiently;
- is adaptable to suit differing local heath service structures within Europe.

These recommendations are in five sections: needs assessment, description of the model, adaptation, standards, and educational implications.

## Development process

The concepts on which these recommendations are based were first explored in a consultation document prepared by the British Association for the Study of Headache [5]. The working group behind that document included secondary-care headache specialists, primary-care physicians with an interest in headache and patient representatives and advocates. The context was, specifically, the National Health Service (NHS) in the United Kingdom; at the time, the NHS was undergoing reorganisation that favoured a general shift of health services from secondary to primary care.

The development group for these recommendations were six headache specialists from Denmark, France, Italy, Spain and United Kingdom. Pre-consultation proposals were published as expert opinions in 2008 [6]. The consultation group included members of the National Headache Societies within the European Headache Federation representing Albania, Belgium, Bulgaria, Denmark, France, Georgia, Germany, Greece, Italy, Norway, Portugal, Romania, Russia, Slovenia, Spain, Switzerland, Turkey and United Kingdom. The consultation process led to revisions and refinements by the development group and, thereby, the production of these recommendations.

## Editorial independence

EHF was the sole funding body supporting development of these recommendations.

## Headache-related health-care needs assessment

This assessment is based on data that exist and on a number of assumptions, which are explained below.

Amongst every 1,000,000 people living in Europe, there are

- 120,000 adults and 15,000 children in need^1^ of professional health care for headache
- requiring the equivalent of 33 doctors working full time in headache medicine.

Population-based studies indicate that amongst every 1,000,000 people living in Europe, there are

- 110,000 adults with migraine [4, 7], 90,000 of whom are significantly disabled [8];
- 600,000 people who have occasional other headaches, the majority being episodic tension-type headache and not significantly disabling;
- 30,000 adults with daily or near-daily headache [4], of whom most are disabled and many have medication-overuse headache.

Existence of a health disorder does not translate directly into need for professional health care. “Need” is generally defined with regard to potential for benefit (there is no need for something that will not be helpful in some way). The proposal that all of the people listed above would gain some benefit from headache care is clearly arguable, but the suggestion that they all have a *need* for care must be constrained in a resource-limited world.

Need predicated on anticipated benefit must rise above a threshold of benefit. Of course this is at the heart of health economics and policy. Thresholds are hard to set objectively, whilst needs assessments are highly sensitive to them. With regard to headache, many people treat themselves, some through necessity, but others from choice. Those who do so are not only those who are less severely affected [8]; many choose self-management when they expect the marginal benefit of professional involvement in their care to be small: sub-threshold benefit negates need. This itself is problematic, because patients’ expectations are quite often unrealistic—either too low or too high—which means that needs assessment based on what people actually do has questionable validity. This is more the case when service improvement is planned: a better service—if “better” means delivering enhanced benefit—should see greater usage than a poor service (“discovered need”). This ought to be factored in, but it cannot readily be estimated.

Aside from these patient-driven highly relevant issues, another is also threshold dependent. Cash-limited health services seek value for money, and will discount needs, however great, whenever utility gain per unit of health-care resource consumption will be low. In headache medicine, this is probably not inequitable: the potential for benefit from professional health care is, generally, greatest amongst those worst affected. Health policy might reasonably focus on these, but perhaps not too restrictively: both migraine and medication-overuse headache are disabling but, in most cases, can be effectively treated at rather low cost whilst mismanagement commonly results in worsening. Health policy should acknowledge this also.

The approach to our needs assessment is conservative: in the face of uncertainty and a number of inestimables described above, it will under-rather than over-estimate need. In the following sections, we set out and explain our assumptions.

### Numbers

A reasonable assumption, we suggest, for the purpose of assessing what should be provided is that only those with disabling headache are in need of professional care. This means, on the basis of the numbers above, 90,000 adults with migraine and 30,000 with daily or near-daily headache: 120,000 adults overall or about 15% of the adult population. There are empirical data from a large UK general practice that support this: 17% of registered patients aged 16–65 years consulted for headache at least once in 5 years [9]. In a Danish population-based study, 11% of adults had consulted a doctor within the last year because of headache [10].

For the child population, need is more difficult to quantify because there are fewer data. Headache is apparently as common in children as in adults, with a 1-year prevalence of >50% [4], but there are different characteristics. It is clear that migraine prevalence is lower in children, dependent upon age, and overall in Europe about half that in adults [4]. On this basis, a reasonable assumption is that, numerically, need for care arises at half the rate per head of that in adults: that is, in 7.5%, or in 15,000 children per 1,000,000 of the general population, where children make up 20% of that population.

### Demand versus need

The issues have been discussed above. On the relationship between “need” (numbers who would benefit from health care) and “demand” (the proportion of those in need who seek health care), complex factors, not all well understood, govern health-care utilisation by people with headache [8]. One is the general lack of availability of care, or its poor quality, which is self-perpetuating until health-care provision is improved. This must be kept in mind, because any assumption about demand is sensitive to this. For the purposes of this assessment, many of the issues discussed earlier are discounted in pursuit of conservatism, and this should be recognised. It is assumed that demand for headache-related health care is expressed by only 50% of those who might be judged to be in need. This has some evidential support [8, 11].

### Time

The need for inpatient management of primary headache is very low. Admission of headache patients with comorbidities, and of patients with medication-overuse headache for detoxication, is sometimes good practice but, overall, fewer than 1% of presenting patients need inpatient care. They can be ignored in these calculations.

The multiple assumptions relating to time allocations, therefore, consider only ambulatory care. They are based on expert views of requirement, again tempered with conservatism.

1. The average consultation need per adult patient is 1.25 h per 2 years. This average is within a wide range of variation, mostly according to diagnosis but also subject to level within the health-care system: consultations in specialist care are usually longer (which may reflect case complexity). In the majority of cases, the total time will be made up of a longer first consultation, including diagnostic enquiry and impact assessment (up to 45 min in specialist care), and 1–3 shorter follow-up appointments in the first and subsequent years.

2. The average consultation need per child patient is greater: 2 h per 2 years. Expert opinion supports this, citing the need for enquiry into family dynamics, schooling and peer relationships as issues relevant to management success.

3. No wastage occurs through failures by patients to attend appointments. This assumption may appear manifestly false, but wastage of this sort is very difficult to predict in the context of proposals for service improvement. At present, such wastage is commonly discounted by overbooking.

4. Each full-time physician (or equivalent) provides 1,344 h of consultation time per year. One day per week is the minimum required for non-clinical work (administration, audit and continuing professional development); each week, therefore, allows 4 days, each of 7 h, of patient-contact time. Only 48 weeks are worked per year.

## Service provision requirement

Despite the conservatism pervading these assumptions, the result is a very challenging estimate of service requirement, expressed in medical full-time equivalents (Table 1). Two conclusions follow.

**Table 1:** Estimated service requirements to meet headache-related health-care demand in a population

Estimated numbers of adults/children with headache care needs per 1,000,000 population	Expected demand (hours of medical consultation per year)
120,000/15,000	45,000 h (33 full-time equivalents)

First, beyond argument, is that most headache services *must* be provided in primary care. This is not a bad thing. Wherever health-care reform is in progress, there is emphasis on strengthening primary care [12]. In addition, and of specific relevance, most headache diagnosis and management requires no more than a basic knowledge of a relatively few very common disorders, which ought to be wholly familiar to primary-care physicians. Only standard clinical skills, which every physician should have, need to be applied. No special investigations or equipment are usually necessary. In other words, there is no good clinical objection to locating most headache services in primary care.

Second, headache services must be formally organised within the structure of local health services generally. If, instead, they merely develop ad hoc, as is currently the case in most of Europe, they cannot possibly be delivered efficiently or equitably.

## A model of headache-service organisation

The fundamental purpose of the model is to divide service provision rationally between primary and secondary (specialist) care. Within a structured health-care system, management of patients at the lowest level commensurate with good care makes most efficient use of allocated resources and is the means by which effective care can reach more who need it. How this is best done clearly depends on the local general health-service structure and on the resources allocated.

However, it also depends on the percentage of presenting patients whose health-care needs cannot be met at primary-care level because of diagnostic or management complexity. Our expert estimate is that 10% of presenting patients might appropriately be treated at a higher level. There are empirical data to support this from a UK general practice: of the adult patients consulting for headache, 9% over a period of time were referred to secondary care [9].

We believe that not all of these require the highest levels of expertise, which is most likely to be available in academic specialist centres. In most countries these are few in number, and they would be overwhelmed if required to manage 10% of patients. We do not believe this is necessary: 1–2% is more realistic.

Accordingly, we recommend the following organisational model (Table 2), and believe it to be suitable for most European countries. As well as proposing services delivered on three interdependent levels, the model sets what are intended as minimum standards; these may be adapted in accordance with local national health service structure, organisation and delivery.

**Table 2:** Headache services organised on three levels

*Level 1.* General primary care	• Frontline headache services (accessible first contact for most people with headache)
	• Ambulatory care delivered by primary health-care providers
	• Referring when necessary, and acting as gatekeeper, to:
*Level 2.* Special-interest headache care	• Ambulatory care delivered by physicians with a special interest in headache
	• Referring when necessary to:
*Level 3.* Headache specialist centres	• Advanced multidisciplinary care delivered by headache specialists in hospital-based centres

## Level 1: General primary care

Non-specialist health-care providers in primary care should meet all of the needs of about 90% (see argument above) of people consulting for headache. At this level, most cases of migraine or tension-type headache should be competently diagnosed and managed. Other common primary and secondary headache disorders listed as core diagnoses (Table 3) should be recognised, but not necessarily managed. Referral channels to levels 2 and 3 should be in place for these cases and for patients who are diagnostically complex or difficult to manage.

**Table 3:** ICDH-II core diagnoses to be recognised at level 1 [13]

Primary headache disorders
1.1 Migraine without aura
1.2 Migraine with aura
1.2.3 Typical aura without headache
2.1 Infrequent episodic tension-type headache
2.2 Frequent episodic tension-type headache
2.3 Chronic tension-type headache
3.1.1 Episodic cluster headache
3.1.2 Chronic cluster headache
Secondary headache disorders
5.2.1 Chronic post-traumatic headache attributed to moderate or severe head injury
6.2.2 Headache attributed to subarachnoid haemorrhage
6.4.1 Headache attributed to giant cell arteritis
7.4.1 Headache attributed to increased intracranial pressure or hydrocephalus caused by neoplasm
8.2 Medication-overuse headache (and subtypes)
9.1 Headache attributed to intracranial infection
10.3 Headache attributed to arterial hypertension
11.3.1 Headache attributed to acute glaucoma
13.1.1 Classical trigeminal neuralgia

On the assumptions above, one full-time practitioner can provide headache care at level 1 for a population no larger than 35,000.

## Level 2: Special interest headache care

Physicians at this level must offer “special interest” services, providing more advanced care to about 10% of patients who are seen at level 1 and referred upwards. Their competence should embrace diagnosis and management of more difficult cases of primary headache and some secondary headache disorders (Table 3), but not those that are very rare. To fulfil their role, they will need access to other services such as neurology, psychology and physiotherapy; for perhaps 10% of their patients they will require a referral channel to level 3.

One full-time physician can provide headache care at level 2 for a population no larger than 200,000.

## Level 3: Headache specialist centres

These centres are likely to be academic. Expert physicians at level 3 should provide advanced care to about 1% of patients first seen at level 1 and referred upwards—either via level 2 or directly, and urgently when necessary. Level 3 should be supported by specialist neurological expertise, have full-time inpatient facilities (with a recommended minimum of two beds per million population) and access to equipment and specialists in other disciplines for diagnosis and management of the underlying causes of all secondary headache disorders, and it should concentrate experience in treating rare headache disorders such as the less-common trigeminal-autonomic cephalalgias.

Level 3 should support levels 1 and 2 through medical advice and education.

One full-time physician can provide headache care at level 3 for a population no larger than 2,000,000.

## The gatekeeper role within the model

The model’s essential purpose is to shift demand from secondary-care services and move it to primary care—a move which in general is cost saving [14]. The *gate*-*keeper* role of primary care [15, 16] is a key issue: the model will not be workable if this role is not embedded at level 1, and patients are allowed to go directly to higher levels regardless of need.

More needs to be said on this. Unrestricted access to specialists induces a demand for costly and sometimes unnecessary services. Patients cannot be blamed for seeking access directly to those they perceive to be experts. Gate-keeping ostensibly guides patients efficiently and in their best interests through the system according to their needs, not their demands. Whatever may be the supposed purpose, gate-keeping probably contributes substantially to cost containment. More importantly, it is the means of preventing specialist services becoming over-loaded, a situation that denies specialist access to some who really need it.

The effectiveness of a system that employs gate-keeping [17], and the equity of it, both rely on efficiency at the level interfaces, seams in service continuity where breakdowns can occur readily and detrimentally to patients [18]. There should not be system-created delays or other barriers set against those who do need specialist care. This is why the model calls for interdependence, and facilitated referral channels, between the levels.

## Adaptation

How this model might be implemented in practice depends not only on the quantity of resources allocated to headache services but also upon the general structure of the health service within which these services are accommodated. Adaptation of the model may be appropriate, and is possible in a number of ways.

### Primary versus secondary care

Level 1 must be in primary care; numbers demand it, and other arguments to support this are expressed earlier. Level 3 centres equally clearly must be in secondary care (or tertiary care in countries that make this distinction). Level 2, on the other hand, can be in either primary or secondary care. Options range from neurologists or trained but non-specialist physicians in district hospital outpatient departments or in polyclinics to general practitioners with a special interest working in primary care (a popular development in the UK [19]).

### Combined levels

There is no intrinsic reason why one centre cannot provide both levels 2 and 3 care. This should not replace any part of level 2 with level 3: this would result in loss of efficiency.

Level 1, by its nature, is or should be community based. It is possible nonetheless, and may be appropriate, for certain level 2 centres to offer, in addition, local level 1 care.

### Division of caseload

The 90:9:1% split between levels 1, 2 and 3 are estimates of need in Europe as a whole, based on expert opinion.

Throughout Europe, there are variations in prevalences and characteristics of the common headache disorders [4], particularly the frequency of daily or near-daily headache [20, 21]. The division of caseload between levels may need some adjustment in particular countries. The model will accommodate this without fundamental change, but capacity at each level will need adjustment. Ideally this would be based on locally gathered empirical data.

### Doctors versus other health-care providers

The model envisages doctor-provided services as the norm at level 1 and as essential at levels 2 and 3. Some countries in Europe are expanding the roles of other professionals in health care as policy. Where this is so, it may allow service delivery at level 1 by nurses or, where they exist, clinical officers trained medically but to a lower level than doctors.

The desirability of this is uncertain, but it is probably a good way forward if the alternative is nothing. Nurses by training are not diagnosticians, but that can be addressed by training. Nurses appear to be very good at follow-up in countries where they are permitted to undertake this role.

## Standards

The following are recommendations as minima.

At level 1, physicians, physician-supervised nurses or clinical officers should:

- have completed a postgraduate theoretical training course in headache medicine;
- have the skills and competencies to diagnose and manage most patients with migraine with or without aura or episodic tension-type headache, following national or EHF guidelines [22];
- recognise other primary and secondary headache disorders listed as core diagnoses (Table 3);
- maintain their skills by practising headache medicine for half a day or more per week on average.

At level 2, physicians should

- acquire their expertise by completing a theoretical and practical training course in headache medicine,
- have the skills and competencies to diagnose and manage more difficult cases of primary headache (all migraine; frequent episodic and chronic tension-type headache; cluster headache and other trigeminal-autonomic cephalalgias) and some secondary headache disorders (chronic post-traumatic headache attributed to moderate or severe head injury; headache attributed to giant cell arteritis; all subtypes of medication-overuse headache; classical trigeminal neuralgia);
- use ICHD-II [13] in their practice;
- follow national or EHF management guidelines [22];
- maintain their skills by practising headache medicine on two days or more per week and by continuing training through regular contact with a level-3 headache centre.

At level 3, specialist physicians should:

- acquire their expertise by:
  - completing a residency programme attached to a level-3 headache centre over one year full-time (or equivalent); and
  - diagnosing and managing 1,000 unselected patients presenting to level 3, with a documented practice record; and
  - making at least two research presentations to national or international conferences and at least two educational lectures;
- apply a multidisciplinary approach in their practice, making use of equipment and specialists in other disciplines in order to diagnose and manage the underlying causes of all secondary headache disorders;
- maintain their skills by:
  - practising headache medicine on two days or more per week; and
  - carrying out or supporting research, and publishing;
- provide formal teaching in headache medicine.

## Educational implications

It is crucial that better knowledge of headache and the use of evidence-based guidelines [22] in primary care keep the great majority of patients at level 1, reducing unnecessary demand upon specialist care. A similar requirement exists at level 2. There are major implications for training.

These need careful consideration. The start, although it is not easily achieved, is to give more emphasis to headache diagnosis and management in the medical schools undergraduate curriculum. This will ensure at least that newly qualified doctors will have some understanding of a set of burdensome and very common disorders—which is often not the case now. However, much more is needed beyond that, and more quickly. The EHF headache schools offer a theoretical and practical course meeting the initial training requirements of level 2 [23]. The Master’s Degree course in headache medicine at Sapienza University, Rome [24, 25], offers a more advanced training-the-trainers course, but has even less reach. Training at national level has to be part and parcel of effective headache-service reform. The educational challenge is greatest at level 1, because of the weight of numbers of health-care providers who need training. Within the 3-level care system proposed, a training role for each higher level to the level below can be envisaged. It is likely that the entire structure will depend on these roles being developed.

## Footnotes

1 “Need” is defined here as existing only in those who are expected to seek access to professional headache care, when available, *and* are likely to benefit.
